# Predictive Value of Pyroptosis Markers (NLRP3, HMGB1, and Caspase‐1) in the Prognosis of Patients With Metastatic Breast Cancer: A Prospective Observational Study

**DOI:** 10.1155/tbj/8821116

**Published:** 2025-12-30

**Authors:** Yi Zhang, Ying He, Qiang Liu, Hongwu Deng

**Affiliations:** ^1^ The Second Department of Breast Surgery, Hunan Cancer Hospital, Affiliated Cancer Hospital of Xiangya Medical School, Central South University, Changsha, 410013, Hunan, China, csu.edu.cn

**Keywords:** caspase-1, HMGB1, metastatic breast cancer, NLRP3, prognosis, pyroptosis, serum biomarkers

## Abstract

**Objective:**

The predictive value of serum pyroptosis markers, including NOD‐like receptor family pyrin domain containing 3 (NLRP3), high mobility group box 1 (HMGB1), and caspase‐1, in metastatic breast cancer (MBC) patients was investigated.

**Methods:**

A prospective observational study was conducted, enrolling MBC patients who had not undergone prior radiotherapy or chemotherapy. Serum levels of pyroptosis markers (NLRP3, HMGB1, and caspase‐1) and conventional tumor markers, including carcinoembryonic antigen (CEA), cancer antigen 125 (CA125), and cancer antigen 15‐3 (CA15‐3), were measured using enzyme‐linked immunosorbent assay (ELISA) kits. Demographic and clinical data were collected, including age, tumor size, hormone receptor status, and metastatic sites. Patients were followed up for 24 months, and overall survival (OS) was recorded.

**Results:**

The study included 122 MBC patients, categorized into the favorable and poor prognosis groups based on 24‐month survival. Elevated levels of NLRP3 and HMGB1 were significantly associated with poor prognosis, whereas lower levels of caspase‐1 were observed in patients with unfavorable outcomes. Receiver operating characteristic (ROC) curve analysis demonstrated that pyroptosis markers, particularly caspase‐1, had significant predictive value for MBC prognosis. Kaplan–Meier curves showed that patients with high HMGB1 levels had a shorter 24‐month OS, whereas those with low caspase‐1 levels also exhibited a shorter 24‐month OS. Multivariate logistic regression analysis identified tumor diameter, Ki67, molecular subtype, number of metastatic sites per patient, and HMGB1 were independent risk factors for poor prognosis in MBC patients, whereas caspase‐1 was an independent protective factor against poor prognosis.

**Conclusion:**

Serum pyroptosis markers (HMGB1 and caspase‐1) were valuable prognostic indicators in MBC patients. Elevated HMGB1 levels, along with reduced caspase‐1 levels, were associated with poorer outcomes.

## 1. Introduction

Breast cancer is among the most prevalent malignancies globally and a leading cause of cancer‐related deaths in women [1, 2]. As reported in Global Cancer Statistics 2020, it has surpassed lung cancer as the most common cancer, accounting for 2.3 million new cases (11.7%) [3]. Despite advances in diagnosis and treatment, metastatic breast cancer (MBC) remains a major clinical challenge, with cancer cells spreading to distant organs such as bones, liver, lungs, or brain, leading to poor prognosis and limited survival [4, 5]. The 5‐year survival rate for MBC is significantly lower than for localized cases, underscoring the need for better prognostic tools and therapies [6–8]. Current treatment options, including chemotherapy, targeted therapy, and immunotherapy, often provide limited benefits due to tumor heterogeneity and acquired resistance [9–11]. Therefore, identifying reliable biomarkers to predict prognosis and guide personalized treatment is critical for improving clinical outcomes in MBC patients.

Serum biomarkers have emerged as valuable tools for cancers [12, 13]. In breast cancer, carcinoembryonic antigen (CEA), cancer antigen 125 (CA125), and cancer antigen 15‐3 (CA15‐3) are widely used, but their predictive value remains suboptimal [14]. Recent studies suggest that pyroptosis, a type of programmed cell death involving membrane rupture and inflammatory cytokine release, plays a role in breast cancer progression and treatment response [15–17]. Pyroptosis is regulated by key molecules, including NOD‐like receptor family pyrin domain containing 3 (NLRP3), high mobility group box 1 (HMGB1), and caspase‐1, which are involved in inflammatory signaling and tumor microenvironment modulation [18–21]. In breast cancer, dysregulation of pyroptosis‐related markers has been linked to tumor aggressiveness, metastasis, and poor prognosis [22]. However, the prognostic value of these markers in MBC patients remains underexplored.

NLRP3 acts as the upstream inflammasome sensor that initiates signal recognition, caspase‐1 functions as the key executor responsible for membrane rupture and maturation of inflammatory cytokines, and HMGB1 serves as a late‐stage alarmin released from ruptured cells that amplifies inflammation [23–25]. This biological chain provides a comprehensive and concise explanation of the pyroptotic process. Therefore, this study focused on the canonical pyroptosis cascade and aimed to investigate the predictive value of serum pyroptosis markers (NLRP3, HMGB1, and caspase‐1) in MBC patients. By analyzing the serum levels of these markers and their association with clinical outcomes, we sought to identify novel prognostic indicators that can complement existing tools for risk stratification and treatment decision‐making. Our findings might contribute to the development of more effective treatment strategies for this challenging disease.

## 2. Methods

### 2.1. Subjects

This prospective observational study enrolled 122 patients diagnosed with MBC from our hospital between January 2017 and December 2022. The diagnosis of MBC was confirmed based on histopathological examination of primary or metastatic lesions, demonstrating invasive breast carcinoma, and imaging studies confirming the presence of distant metastases. Metastatic lesions were defined as measurable disease according to the Response Evaluation Criteria in Solid Tumors (RECIST) version 1.1 [26]: Metastatic lesions were confirmed by the presence of at least one target lesion ≥ 10 mm in the longest diameter for nonlymph nodes or ≥ 15 mm in the short axis for lymph nodes. The diagnosis and staging were performed in accordance with the National Comprehensive Cancer Network (NCCN) guidelines [27] for breast cancer, which include histological confirmation, imaging evidence of metastasis, and clinical assessment of disease extent.

The inclusion criteria were as follows: (1) age ≥ 18 years; (2) histologically or cytologically confirmed MBC; (3) presence of measurable metastatic lesions as defined by the RECIST version 1.1; and (4) no prior radiotherapy or chemotherapy before enrollment. The exclusion criteria were as follows: (1) history of other malignancies within the past 5 years; (2) severe comorbidities, such as end‐stage renal disease, decompensated liver cirrhosis, or uncontrolled cardiovascular diseases, that could interfere with the study outcomes; (3) incomplete clinical data; (4) male patients; and (5) loss to follow‐up. All patients underwent tumor resection according to the MBC treatment guidelines of our hospital, and the follow‐up period for all enrolled patients was set at 24 months to ensure comprehensive evaluation of prognostic outcomes. This study was approved by the Ethics Committee of Hunan Cancer Hospital (No. HCH2017014), and all participants provided informed consent to participate in the study.

### 2.2. Measurement of Serum Pyroptosis Markers and Tumor Markers

To evaluate the expression levels of pyroptosis‐related markers and tumor markers, peripheral blood samples (approximately 5 mL per patient) were collected from all enrolled MBC patients before the initiation of any treatment. Serum was separated by centrifugation at 3000 rpm for 10 min and stored at −80°C. The concentrations of pyroptosis markers, including NLRP3 (MBS167322, detection range 2–600 ng/L, intra‐assay CV < 8%, and interassay CV < 10%; MyBioSource, USA), HMGB1 (MBS3804267, detection range 3.125–50 ng/mL, intra‐assay CV < 10%, and interassay CV < 10%; MyBioSource, USA), and caspase‐1 (MBS264676, detection range 0.312–20 ng/mL, intra‐assay CV < 8%, and interassay CV < 12%; MyBioSource, USA), were measured using commercially available enzyme‐linked immunosorbent assay (ELISA). All samples were tested in duplicate, and the average values were recorded to ensure accuracy. In addition to pyroptosis markers, CA15‐3, CEA, and CA125 were also assessed. The measurements of these tumor markers were performed using standardized immunoassay techniques. The results were analyzed to establish baseline levels and explore potential correlations with pyroptosis markers and clinical outcomes.

### 2.3. Clinical Data

Comprehensive demographic and clinical data were collected for all enrolled MBC patients to evaluate their potential impact on prognosis. Demographic information included age, body mass index (BMI), and menopausal status. Clinical characteristics encompassed tumor size, Ki67, histological type, tumor node metastasis (TNM), and molecular subtype. Additionally, data on metastatic sites and the number of metastatic lesions were recorded.

### 2.4. Follow‐Up

The overall survival (OS) of all MBC patients was recorded, and patients were categorized into the favorable or poor prognosis groups according to whether they died or not. All enrolled patients were followed up for a period of 24 months to evaluate clinical outcomes. The primary endpoint of the study was OS, defined as the time from the date of enrollment to the date of death from any cause or the last follow‐up. Patients were regularly monitored through clinical visits or telephone interviews at 3‐month intervals. During each follow‐up, survival status, disease progression, and any additional treatments received were recorded. Based on their survival status at the end of the follow‐up period, patients were categorized into two groups: the favorable prognosis group (patients who survived) and the poor prognosis group (patients who died).

### 2.5. Statistical Analysis

Statistical analyses were performed using SPSS software (version 26.0). Continuous variables were presented as mean ± standard deviation (SD) or median with interquartile range (IQR) based on their distribution. Categorical variables were expressed as frequencies and percentages. The Mann–Whitney test or Student’s *t*‐test was used for comparisons between two groups. The chi‐square test analyzed ratios, whereas Pearson’s correlation assessed associations. Receiver operating characteristic (ROC) curves evaluated serum pyroptosis markers for prognosis. Kaplan–Meier (K–M) curves estimated OS, and logistic regression identified risk factors for poor prognosis.

## 3. Results

### 3.1. Clinical Characteristics

A total of 122 MBC patients were followed up for 24 months and categorized into two groups based on their survival status: the favorable prognosis group (patients who survived, *n* = 71) and the poor prognosis group (patients who died, *n* = 51). The results indicate that patients with a poor prognosis exhibited significantly larger tumor diameters and higher Ki67 levels. Additionally, the proportions of patients with liver metastases, lung metastases, and multiple (≥ 2) metastatic sites were significantly higher in the poor prognosis group than in the favorable prognosis group (Table 1, *p* < 0.05).

**Table 1:** Clinical characteristics of metastatic breast cancer patients.

Variable	Poor prognosis group, *n* = 51	Favorable prognosis group, *n* = 71	*p*
Age, y	57 (44–71)	56 (41–68)	0.193
BMI	22.13 ± 2.40	22.42 ± 2.21	0.483
Menopausal status			0.378
Premenopausal, *n* (%)	17 (33.3)	28 (39.4)	
Postmenopausal, *n* (%)	34 (66.7)	43 (60.1)	
Tumor diameter (cm)	4.57 ± 1.28	3.29 ± 1.17	< 0.001
Ki67 (%)	42.36 ± 11.03	32.05 ± 8.98	< 0.001
Molecular subtype			0.067
HR+/HER2−, *n* (%)	30 (58.8)	53 (74.6)	
HER2+, *n* (%)	7 (13.7)	10 (14.1)	
TNBC, *n* (%)	14 (27.5)	8 (11.3)	
Metastatic site			
Bone, *n* (%)	6 (11.8)	14 (19.7)	0.125
Liver, *n* (%)	32 (62.7)	26 (36.6)	< 0.001
Lung, *n* (%)	34 (66.7)	32 (45.1)	0.002
Brain, *n* (%)	8 (15.7)	13 (18.3)	0.625
Number of metastatic sites per patient			< 0.001
1 site, *n* (%)	25 (49.0)	58 (81.7)	
≥ 2 sites, *n* (%)	26 (51.0)	13 (18.3)	

*Note:* Hormone receptor‐positive/HER2‐negative (HR+/HER2−); HER2‐positive (HER2+).

Abbreviations: BMI, body mass index; TNBC, triple‐negative breast cancer.

### 3.2. Serum Levels of Pyroptosis Markers in MBC Patients

**Figure 1:**
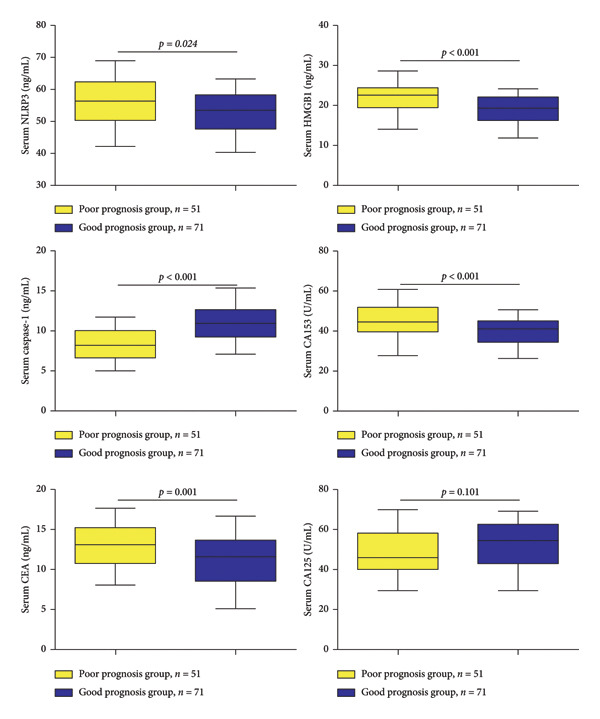
Serum levels of pyroptosis markers in MBC patients. In boxplots, data are presented as median (line within the box), interquartile range (box indicating 25th–75th percentiles), and minimum to maximum (whiskers). The normality of continuous variables was assessed using the Shapiro–Wilk test, and all data were confirmed to be normally distributed. Comparisons between two groups were performed using Student’s *t*‐test. NOD‐like receptor family pyrin domain containing 3 (NLRP3), high mobility group box 1 (HMGB1), cancer antigen 15‐3 (CA15‐3), carcinoembryonic antigen (CEA), and cancer antigen 125 (CA125).

We determined the serum levels of NLRP3, HMGB1, caspase‐1, CA15‐3, CEA, and CA125 in all patients. As shown in Figure 1, between the favorable and poor prognosis groups, the levels of the cellular pyrogenic factors HMGB1 and NLRP3 were remarkably elevated in the poor prognosis group, whereas caspase‐1 was markedly decreased. In addition, for conventional tumor markers, the poor prognosis group showed markedly enhanced serum levels of CA15‐3 and CEA compared to the favorable prognosis group. Moreover, a weak but statistically significant positive correlation was observed between CA15‐3 and HMGB1 (*r* = 0.192 and *p* = 0.034) as well as a weak negative correlation between CA15‐3 and caspase‐1 (*r* = −0.286 and *p* = 0.001; Table 2).

**Table 2:** Correlation analysis between pyroptosis and tumor markers.

Variables		NLRP3	HMGB1	Caspase‐1
CA15‐3	Pearson’s correlation	0.171	0.192	−0.286
	*P*	0.059	0.034	0.001

CEA	Pearson’s correlation	−0.002	0.145	−0.142
	*P*	0.984	0.110	0.119

CA125	Pearson’s correlation	−0.108	−0.025	0.006
	*P*	0.237	0.783	0.951

*Note:* NOD‐like receptor family pyrin domain containing (NLRP), cancer antigen 15‐3 (CA15‐3), and cancer antigen 125 (CA125).

Abbreviations: CEA, carcinoembryonic antigen; HMGB1, high mobility group box 1.

### 3.3. Predictive Value of Pyroptosis Markers for Prognosis in MBC Patients

**Figure 2:**
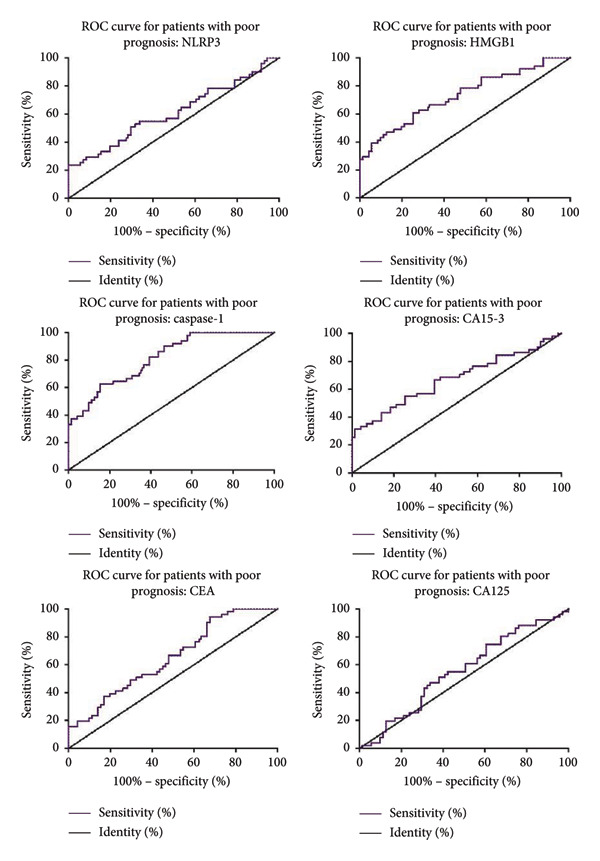
ROC curves of pyroptosis markers for predicting prognosis in MBC patients. NOD‐like receptor family pyrin domain containing 3 (NLRP3), high mobility group box 1 (HMGB1), cancer antigen 15‐3 (CA15‐3), carcinoembryonic antigen (CEA), and cancer antigen 125 (CA125).

**Figure 3:**
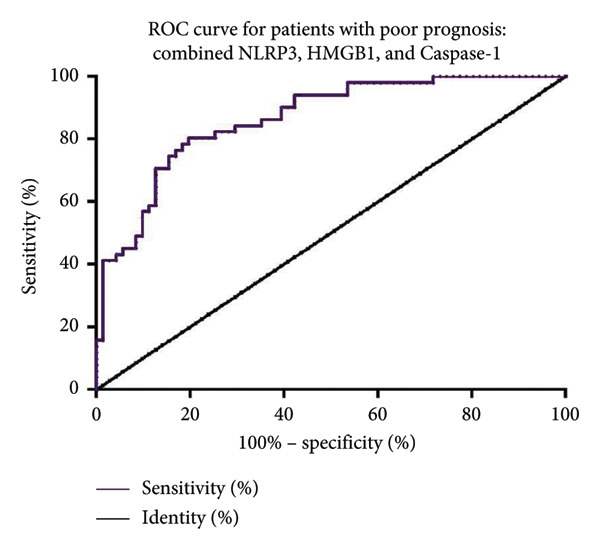
ROC curve of the combined NLRP3, HMGB1, and caspase‐1 model for predicting prognosis in MBC patients. NOD‐like receptor family pyrin domain containing 3 (NLRP3) and high mobility group box 1 (HMGB1).

We further analyzed the predictive value of serum pyroptosis marker levels on the prognosis of MBC patients using ROC curves. As shown in Figure 2, for predicting poor prognosis in MBC patients, NLRP3 had an AUC of 0.609 (95% CI: 0.505–0.714, cutoff: 55.53 ng/mL, sensitivity: 54.9%, and specificity: 66.2%) and HMGB1 had an AUC of 0.728 (95% CI: 0.636–0.820, cutoff: 21.14 ng/mL, sensitivity: 66.7%, and specificity: 66.2%). Caspase‐1 showed the highest predictive value with an AUC of 0.813 (95% CI: 0.740–0.887, cutoff: 9.33 ng/mL, sensitivity: 71.8%, and specificity: 66.7%). In addition, CA15‐3 showed an AUC of 0.671 (95% CI: 0.569–0.772, cutoff: 41.85 U/mL, sensitivity: 66.7%, and specificity: 60.6%), CEA had an AUC of 0.650 (95% CI: 0.553–0.747, cutoff: 12.29 ng/mL, sensitivity: 58.8%, and specificity: 54.9%), and CA125 had an AUC of 0.554 (95% CI: 0.451–0.657, cutoff: 50.70 U/mL, sensitivity: 57.7%, and specificity: 54.9%). Furthermore, the combined NLRP3, HMGB1, and caspase‐1 model demonstrated superior predictive performance, with an AUC of 0.865 (95% CI: 0.801–0.928, cutoff: 0.58, sensitivity: 80.3%, and specificity: 80.4%) (Figure 3).

### 3.4. K–M Curve Analysis

**Figure 4:**
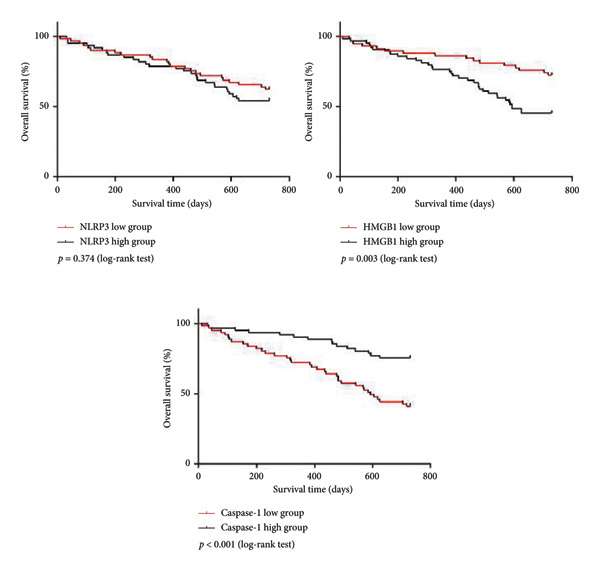
Kaplan–Meier curves for survival analysis of pyroptosis markers in MBC patients. NOD‐like receptor family pyrin domain containing 3 (NLRP3) and high mobility group box 1 (HMGB1).

Then, we assessed the 24‐month survival of MBC patients using the K–M curve analysis. The mean NLRP3 level was set at 54.01 ng/mL, the mean HMGB1 level was set at 20.28 ng/mL, and the mean caspase‐1 level was set at 9.85 ng/mL. The results showed that patients with high HMGB1 levels had a shorter 24‐month OS, whereas those with low caspase‐1 levels also exhibited a shorter 24‐month OS (Figure 4, *p* < 0.05).

### 3.5. The Risk Factors for Poor Prognosis in MBC Patients

Finally, all clinically relevant variables were entered simultaneously into the multivariate binary logistic regression model to analyze risk factors for poor prognosis in MBC patients. The results showed that tumor diameter (OR = 2.184 and 95% CI: 1.095–4.356), Ki67 (OR = 1.138 and 95% CI: 1.029–1.259), molecular subtype (OR = 4.370 and 95% CI: 1.486–12.849), number of metastatic sites per patient (OR = 17.633 and 95% CI: 2.884–107.817), and HMGB1 (OR = 1.461 and 95% CI: 1.114–1.917) were independent risk factors for poor prognosis in MBC patients, whereas caspase‐1 (OR = 0.501 and 95% CI: 0.314–0.801) was an independent protective factor against poor prognosis (Table 3).

**Table 3:** Multivariate logistic regression analysis of risk factors for poor prognosis in MBC patients.

Variables	Odds ratio	95% CI	*p*
Age, y	1.035	0.949–1.128	0.436
BMI	0.831	0.601–1.149	0.264
Menopausal status	1.790	0.395–8.115	0.450
Tumor diameter	2.184	1.095–4.356	0.026
Ki67	1.138	1.029–1.259	0.012
Molecular subtype	4.370	1.486–12.849	0.007
Number of metastatic sites per patient	17.633	2.884–107.817	0.002
NLRP3	1.056	0.948–1.177	0.319
HMGB1	1.461	1.114–1.917	0.006
Caspase‐1	0.501	0.314–0.801	0.004
CA15‐3	1.035	0.945–1.135	0.457
CEA	1.208	0.912–1.602	0.188
CA125	0.933	0.861–1.010	0.086

*Note:* NOD‐like receptor family pyrin domain containing (NLRP) and cancer antigen 15‐3 (CA15‐3). All clinically relevant variables were entered simultaneously into the multivariate logistic regression model.

Abbreviations: BMI, body mass index; CA125, cancer antigen 125; CEA, carcinoembryonic antigen; HMGB1, high mobility group box 1; TNBC, triple‐negative breast cancer.

## 4. Discussion

Epidemiological reports estimate a 54.8% increase in the number of MBC patients by 2030 compared to 2015, which will create a huge physical and economic burden for society and individuals [28, 29]. Despite advancements in systemic therapies, the prognosis for MBC patients remains poor, with a median survival of only 2–3 years [30]. This underscores the critical need for reliable prognostic tools to guide treatment decisions and improve patient outcomes. In this study, we investigated the prognostic value of serum pyroptosis markers (NLRP3, HMGB1, and caspase‐1) in MBC patients. Our findings demonstrated that elevated levels of HMGB1, along with reduced levels of caspase‐1, were significantly associated with poor prognosis.

The identification of prognostic risk factors is essential for optimizing treatment strategies in MBC. Traditional clinical and pathological factors (tumor size, metastatic sites, and conventional tumor markers like CA15‐3, CEA, and CA125) have long been used to predict outcomes [31]. However, these factors alone are often insufficient to capture the heterogeneity of MBC. For example, CA15‐3, although widely used, often fails to provide early prognostic information, and CEA is not specific to breast cancer [32]. In recent years, serum biomarkers have gained attention due to their noninvasive nature, ease of measurement, and potential to reflect real‐time tumor dynamics. Several studies have explored novel serum markers to improve prognostic accuracy in breast cancer. For instance, semaphorin 4C (SEMA4C) has shown promising diagnostic potential for breast cancer, with elevated serum levels significantly associated with tumor presence and reduced levels postsurgery, suggesting its utility in monitoring disease progression [33]. Similarly, selenium status biomarkers, including total glutathione peroxidase 3 (tGPX3) activity, have been linked to improved OS and reduced recurrence in breast cancer patients, highlighting the prognostic value of trace elements in cancer biology [34]. Additionally, serum exosomal long noncoding RNA (lncRNA) XIST is a potential biomarker for triple‐negative breast cancer (TNBC) recurrence, with higher levels linked to poorer OS [35]. In our study, ROC curve analysis demonstrated that pyroptosis markers (NLRP3, HMGB1, and caspase‐1) could effectively predict the prognosis of MBC patients, further supporting the potential of serum biomarkers in enhancing risk stratification and treatment decision‐making.

Pyroptosis, an inflammatory form of programmed cell death involving membrane rupture, has recently gained attention in cancer biology [15]. The pyroptosis‐related markers NLRP3, HMGB1, and caspase‐1 are known to regulate inflammatory signaling and modulate the tumor microenvironment [36]. In breast cancer, Saponaro et al. found that patients with higher NLRP3 expression in tumor tissues had worse 5‐year disease‐free survival (DFS) and poorer prognosis compared to those with lower NLRP3 expression [37]. Peng et al. showed that caspase‐1 expression in BC tumor tissues was lower than in surrounding tissues, and patients with high caspase‐1 expression had better OS, disease‐specific survival (DSS), and progression‐free interval (PFI) than patients with low caspase‐1 expression [38]. Chen et al. showed in basic research that HMGB1 secreted by breast cancer cells activates fibroblasts through receptor for advanced glycation end products (RAGEs)/aerobic glycolysis, and these activated fibroblasts promote cancer cell metastasis by increasing lactate production [39]. These findings suggested that NLRP3 and HMGB1 might drive MBC progression, whereas caspase‐1 may exert a protective effect, highlighting the complex role of pyroptosis in cancer biology.

The mechanisms underlying the association between pyroptosis markers and MBC prognosis are likely multifaceted. NLRP3, a key component of the inflammasome complex, is known to promote inflammation and tumor progression by activating proinflammatory cytokines [40]. Elevated NLRP3 levels in the poor prognosis group may reflect a more aggressive tumor phenotype characterized by enhanced inflammation and immune evasion. HMGB1, a damage‐associated molecular pattern (DAMP) molecule, has been shown to promote tumor metastasis by modulating the tumor microenvironment and facilitating angiogenesis [41]. In contrast, caspase‐1, which cleaves and activates proinflammatory cytokines, may play a dual role in cancer. Although its proinflammatory functions can contribute to tumor progression, its role in inducing pyroptosis may also suppress tumor growth [19]. Caspase‐1 is a central executor of inflammasome activation, mediating gasdermin‐dependent pyroptosis and the maturation of IL‐1β and IL‐18. Reduced systemic caspase‐1 activity may attenuate pyroptotic cell death and the release of DAMPs, thereby weakening the activation of cytotoxic immune cells and impairing antitumor immune surveillance [42]. This immunosuppressive state might partially account for accelerated tumor progression and unfavorable prognosis. In our study, we observed significantly higher levels of NLRP3 and HMGB1 in the poor prognosis group, whereas caspase‐1 levels were lower in these patients. These findings aligned with previous research and provide a foundation for further investigation into the molecular mechanisms linking pyroptosis to MBC progression.

From a clinical perspective, serum pyroptosis markers such as NLRP3, HMGB1, and caspase‐1 may hold potential value as supplementary prognostic indicators in MBC. The combined assessment of these three markers showed improved predictive performance compared with individual markers, suggesting that a multimarker approach might enhance risk stratification in clinical settings. Incorporating such markers into existing prognostic frameworks could, in the future, help identify patients at higher risk of disease progression or suboptimal treatment response. In addition, given the close association between pyroptosis and the tumor immune microenvironment, these biomarkers may provide preliminary guidance for individualized therapeutic decisions, including the possible selection of patients who could benefit from immunotherapy or combined treatment strategies targeting inflammasome‐related pathways. Nevertheless, further prospective, large‐scale studies are warranted to validate their clinical applicability and determine whether integrating these markers into current prognostic models can meaningfully improve outcome prediction.

Although our study provides valuable insights into the prognostic value of pyroptosis markers in MBC, several limitations should be acknowledged. The sample size was relatively small, which may limit the reliability of subgroup analyses, and treatment heterogeneity might have introduced potential confounding effects. In addition, the absence of an independent validation cohort limits the generalizability of our findings. The follow‐up period of 24 months may not fully capture long‐term survival outcomes. Furthermore, we did not explore the underlying molecular mechanisms linking pyroptosis markers to MBC progression or assess other inflammasome sensors and noncanonical caspases. Future studies should include larger cohorts with external validation and further mechanistic investigations to determine whether additional pathways provide incremental prognostic value beyond the NLRP3 caspase‐1 HMGB1 axis.

## 5. Conclusion

In conclusion, our study demonstrated that elevated levels of HMGB1, along with reduced levels of caspase‐1, were significantly associated with poor prognosis in MBC patients. These findings suggest that pyroptosis markers play a critical role in MBC progression and may serve as valuable prognostic indicators. By incorporating these markers into prognostic models, clinicians may improve risk stratification and personalize treatment strategies for MBC patients, ultimately enhancing clinical outcomes.

## Ethics Statement

This study was approved by the Ethics Committee of Hunan Cancer Hospital (No. HCH2017014), and all participants provided informed consent to participate in the study.

## Consent

Please see the ethics statement.

## Disclosure

All authors reviewed and approved the final version of the manuscript.

## Conflicts of Interest

The authors declare no conflicts of interest.

## Author Contributions

Yi Zhang conceived and designed the study. Ying He, Qiang Liu, and Hongwu Deng performed the experiments. Qiang Liu and Hongwu Deng analyzed the data. Yi Zhang and Ying He wrote the manuscript.

## Funding

The authors received no specific funding for this work.

## Data Availability

The datasets used or analyzed during the current study are available from the corresponding author on reasonable request.
